# Rethinking microbial biopesticide development and uptake

**DOI:** 10.1007/s11274-026-05044-3

**Published:** 2026-05-29

**Authors:** Travis R. Glare, Andy Sheppard, Mark R. H. Hurst, Louise Thatcher, Marta Gallart, Sarina Macfadyen, Simon Law, Maureen O’Callaghan

**Affiliations:** 1 Lincoln Agritech Ltd, Lincoln University, Canterbury, New Zealand; 2 Health and Biosecurity, Commonwealth Scientific and Industrial Research Organisation (CSIRO), Clunies Ross Street, Acton, ACT Australia; 3 Agriculture and Food, Commonwealth Scientific and Industrial Research Organisation (CSIRO), Clunies Ross Street, Acton, ACT Australia; 4 Bioeconomy Science Institute, Tuhiraki, 19 Ellesmere Junction Road, Lincoln, Canterbury, 7608 New Zealand

**Keywords:** Microbial biopesticides, Commercialisation, Decision-support framework, Biocontrol agents, Market driven development, Regulatory pathways, Entomopathogens, Mode of action, Product development pipeline, Uptake and adoption barriers, Sustainable pest management

## Abstract

Biopesticides, pesticides based on living organisms and/or their bioactive compounds, are increasingly being used as alternatives and replacements to chemical and synthetic pesticides. This is largely due to human and environmental safety concerns, the emergence of pest resistance (collectively insect pests, weeds and diseases), and the move towards holistic pest and disease control approaches. While the number and diversity of agents used in biopesticides slowly increases, the approach to developing these products remains largely ad hoc, resulting in less than 10% success rate for development projects. Here we review the major characteristics of successful biopesticides. Our treatise focuses on the benefits of considering the entire development pathway and requirements of the intended product application before embarking on the costly process of biopesticide development and commercialisation. By a priori consideration of the characteristics of both the target pest and market, and the potential limitations of the candidate microorganism and/or its bioactives (e.g. environmental persistence, ease and cost of mass production), the development pipeline can be streamlined and targeted on projects with the greatest likelihood of success. We provide a detailed consideration of the key factors that underpin successful (or not) biopesticide development and provide decision trees to support the a priori process.

## Introduction

Biopesticides are agricultural products based on naturally occurring organisms and compounds that have activity against pests, including invertebrates, plant diseases and weeds. Microbial biopesticides are based on microorganisms - either the whole microorganism (living or dead) or bioactives derived from a microorganism or both (Koul 2011; Collinge et al. 2022) and are the focus of this paper. The use of microorganisms to control insects and plant diseases has a long history, with the first practical use of an insect pathogen *Metarhizium* sp. against a wheat chafer in 1888 (Zimmerman et al. 1995). There was less focus on biopesticides following the advent of highly effective synthetic pesticides in the 1950s, but research interest resurged in the 1980s when the adverse effects of intensive pesticide use (non-target toxicity, long term persistence in the environment and food chain, and pest resistance) became clear. Use of microbial biopesticides has increased only slowly since this time, with the products dogged with perceptions of poor efficacy and lack of understanding of their use. The exception has been products based on the bacterial entomopathogen *Bacillus thuringiensis* (*Bt*), which has been commercially available since 1938 (Glare and O’Callaghan 2000) and for many years since has been the active ingredient in over 90% of all commercial biopesticides. The key to the dominance of *Bt* in the biopesticide market lies in its biology, including its toxins which are active against several pests, and that it does not require live cells for toxicity. In addition, *Bt* can be mass produced cheaply, and products have a long shelf life. As discussed below, consideration of these biological characteristics is critical in identification and development of emerging biopesticides today. It needs to be recognised that *Bt* is the exception rather than the rule for microbes used as actives in biopesticides, as the action is not dependent on a live cell. However, the characteristics that make *Bt* so successful can also serve as a guide for evaluation other microbial control agents in terms of field efficacy, stability, market and safety.

Microbial biopesticides, including microbial control agents for management of plant diseases and weeds, have multiple benefits compared to many synthetic pesticides, such as targeted action and specificity resulting in reduced non-target impacts on beneficial species. Biopesticides can be used to manage pesticide resistance and because of their biodegradability and low residues, they tend to have no (or short) withholding periods, and do not result in water and soil pollution. These benefits over traditional chemical controls make a compelling value proposition to growers (Marrone 2019). In addition, their specific modes of action ensure they are safer for mammals (Fusar et al. 2024; Cai and Dimopoulos 2025). Because of these many favourable characteristics, microbial biopesticides have increasingly become recognized as viable technologies that are acceptable for mainstream use, rather than as niche market products (Ruiu 2018; Boyetchko et al. 2020). They also offer solutions where synthetic pesticides have been withdrawn from market, for example due to resistance development, new or restricted market access, niche or small production crops, or under scenarios requiring short withholding periods. Their market share is now growing as synthetic pesticides are increasingly being legislated against and withdrawn from markets and as public acceptability diminishes with growing environmental and health concerns (Lykogianni et al. 2021). Legislation in the EU has been indicative of the trend towards reduced use of pesticides (Ehlers 2011; Balog et al. 2017), with the proposed 2030 reduction requirement of 50% reduction in synthetic pesticide use (EU 2024). However, this target was recently rejected by the EU parliament (EU 2024) due to the lack of available alternatives, and the use of some controversial synthetic pesticides has now been extended beyond this deadline (Larchet and Guillem 2024; Neve et al. 2024). However, many other countries continue to adopt pesticide reduction targets (Marrone 2024; Fenibo et al. 2021; Jiang et al. 2024). For example, the USA had a drive towards building a vibrant domestic biomanufacturing ecosystem (US Executive order; 12 September 2022) (Karamaouna et al. 2023). Brazil has used a permissive regulatory approach to promote development and availability of biopesticides, such that over 850 products are currently registered (Hirata 2025).

In response to these drivers, the global biopesticide market is currently valued at over US$5B and is expected to grow to US$15B by 2029 (Marrone 2024). This growth rate exceeds that of the synthetic pesticide market where increasing costs and more stringent regulatory processes have slowed the development and release of new chemistries. The steady increase in biopesticide sales reflects the growing need for sustainable and safe pesticides (Ayilara et al. 2023). While there is still a long way to go before biopesticides displace synthetic pesticides, biopesticides now account for around 10% of the global pesticide market (Marrone 2024).

Historically, commercialization of biopesticides was conducted by small to medium sized enterprises (SMEs), with successful SMEs often acquired by multinational companies, seeking to extend their plant protection product portfolios in response to demand from growers for safer products. Today, most multinational agrichemical companies (e.g. Syngenta, Certis) have well tested biopesticides within their product range, with these typically focussed on major crops such as corn, soybean, rice, cotton and potato (Boyetchko 2020). More recently there has been a proliferation of start-up companies based on emerging technologies that would previously have been bought out by the large agrichemical companies, but their high failure rate has led to a model where big players invest in the startups for success rather than buying them out too early (Marrone 2024). While there is renewed interest and investment into biopesticide development, there is still a relatively narrow range of scientifically validated, commercially viable products (indeed none exist for weeds), except perhaps in the context of small focussed and localised markets (Marrone 2024). Despite decades of academic research demonstrating the biopesticidal potential of a wide range of microbes, only a very small minority of these have achieved commercialization and uptake by growers. Many reviews have addressed specific barriers to commercialisation of biopesticides (e.g. Sachdev and Singh 2016; Rijswijk et al. 2018; Koul 2023; Duke 2024). Glare et al. (2012) identified areas that needed improvement including delivery and persistence, advancing the use of bioactives as opposed to whole cells (to overcome issues with poor shelf life) and more strategic selection of target pests and market. Some of these needs have not changed and, despite another decade of scientific research, new scientifically validated biopesticide solutions are still slow to reach the market.

In this review we highlight critical challenges in microbial biopesticide development, aspects of commercialization and on-farm uptake, and guide researchers on how to steer clear of common mistakes and assumptions by knowing your market, your microorganism and your target pest. We propose an alternative product development strategy, identify opportunities for further research and development to reduce cost of goods and improve field efficacy, and highlight the essential “non-science” activities that can make the difference between success and failure. Importantly, we focus on early-stage consideration of aligning market need, the biology of the pest(s) with potential agents. By early consideration of these factors, development time can be reduced and development costs minimized. More comprehensive evaluation of a potential microbial agent, target and market need early in the process can identify likely issues and quickly highlight problems that would prohibit or impede successful commercialization. This will lead to efficient fast-fail screening of multiple candidate microorganisms, or early identification of critical technical challenges that must be addressed to avoid product failure (Verma et al. 2024).

## Approaches to biopesticide development

**Fig. 1:**
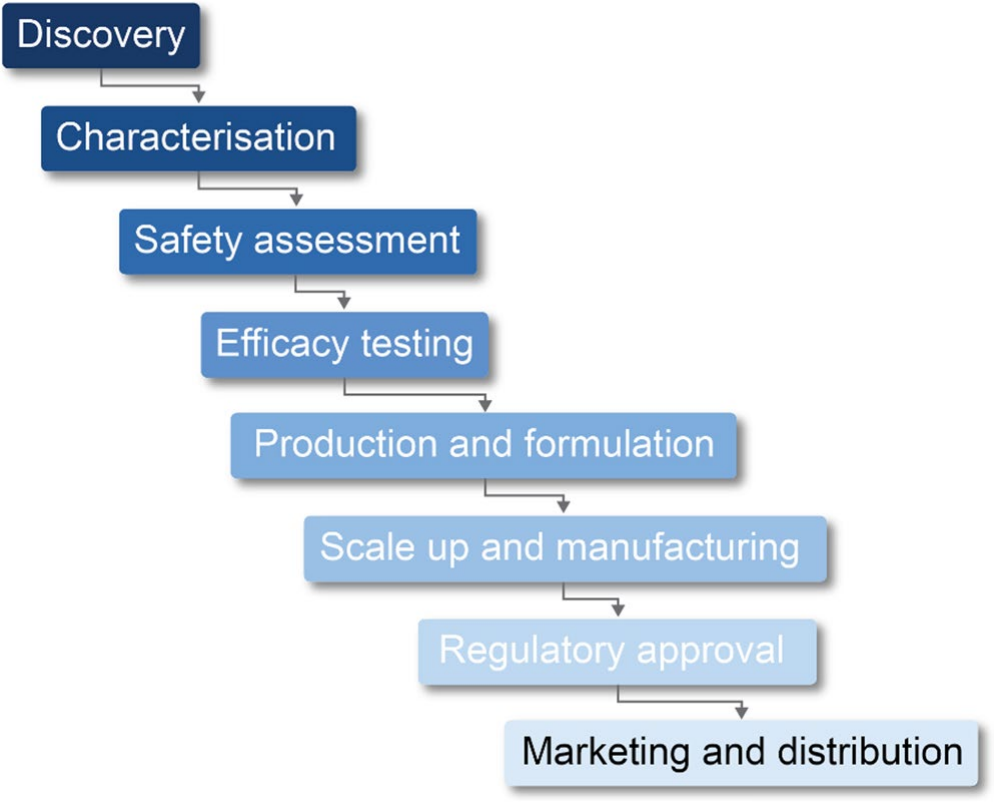
Generalised biopesticide development pipeline

Historically, biopesticide development has followed a linear pipeline approach (Fig. 1), typically beginning with discovery and early research (often within universities and research organisations) on biopesticidal microorganisms. Common starting points for research include: (1) a target pest is selected and a biodiscovery process is used to find a suitable microbial solution; or (2) a novel bioactive microorganism (or bioactive from the microorganism) has been discovered, possibly against non-target pests, and developed through the biopesticide pathway to find a suitable economic target (Mandakini and Manamgoda 2021). Following discovery and initial characterisation of candidate biopesticidal microorganisms, research typically progresses through the stages outlined in Fig. 1. Laboratory and glasshouse evaluation is undertaken next, often yielding promising results. However, subsequent evaluations of field efficacy often fail to match expectations based on laboratory testing (O’Sullivan et al. 2021; Boyetchko 2020). To gather the data needed to protect IP and attract investment from commercial partners, further extensive research is then undertaken to demonstrate and/or improve field efficacy, perhaps by a prolonged effort to improve formulations, for example. In theory, there will be a series of stage-gates or “Go vs. No-Go” criteria to support decisions on the feasibility of developing a viable product, but in practice this does not always happen. Progression through this linear development pipeline has often been driven by researcher-led “science push”, sometimes with myopic focus on a specific microorganism which might be less efficacious than competing products, and without awareness of “commercialization red flags”. The success rate of prototypes emerging from this somewhat academic pipeline approach can be as low as 10% (Hubbard et al. 2014).

With growing industry investment in biopesticide identification and development, sufficient resources have become available to support high throughput mass screening of multiple microbes and their bioactive compounds grown under different conditions against an array of different pests and diseases, a process increasingly enabled by robotic screening platforms, large proprietary collections of microorganisms, and compound libraries. With the increasing use of AI to expedite all stages of the product development pipeline (e.g. Holzinger et al. 2023; Zhu et al. 2025), including predictions of future field efficacy, combined with the use of genomics and multi-omics platforms, it is expected that the success rate for the discovery of new modes of action and microbial agents will increase significantly and reduce the time taken to bring products to market. More importantly, these approaches can be used very early in the agent selection process, helping predict which agents will have the greatest chance of commercial success. However, regardless of the process by which a biopesticidical agent is discovered, there remain multiple reasons why biopesticide prototypes fail to be commercialized and taken up by growers, all of which must be considered early in the development process if costly mistakes are to be avoided. Both technical issues and broader market dynamics contribute to the small market share currently held by biopesticides (Poli and Fontebrancesco 2024).

## Addressing factors critical to success

**Fig. 2:**
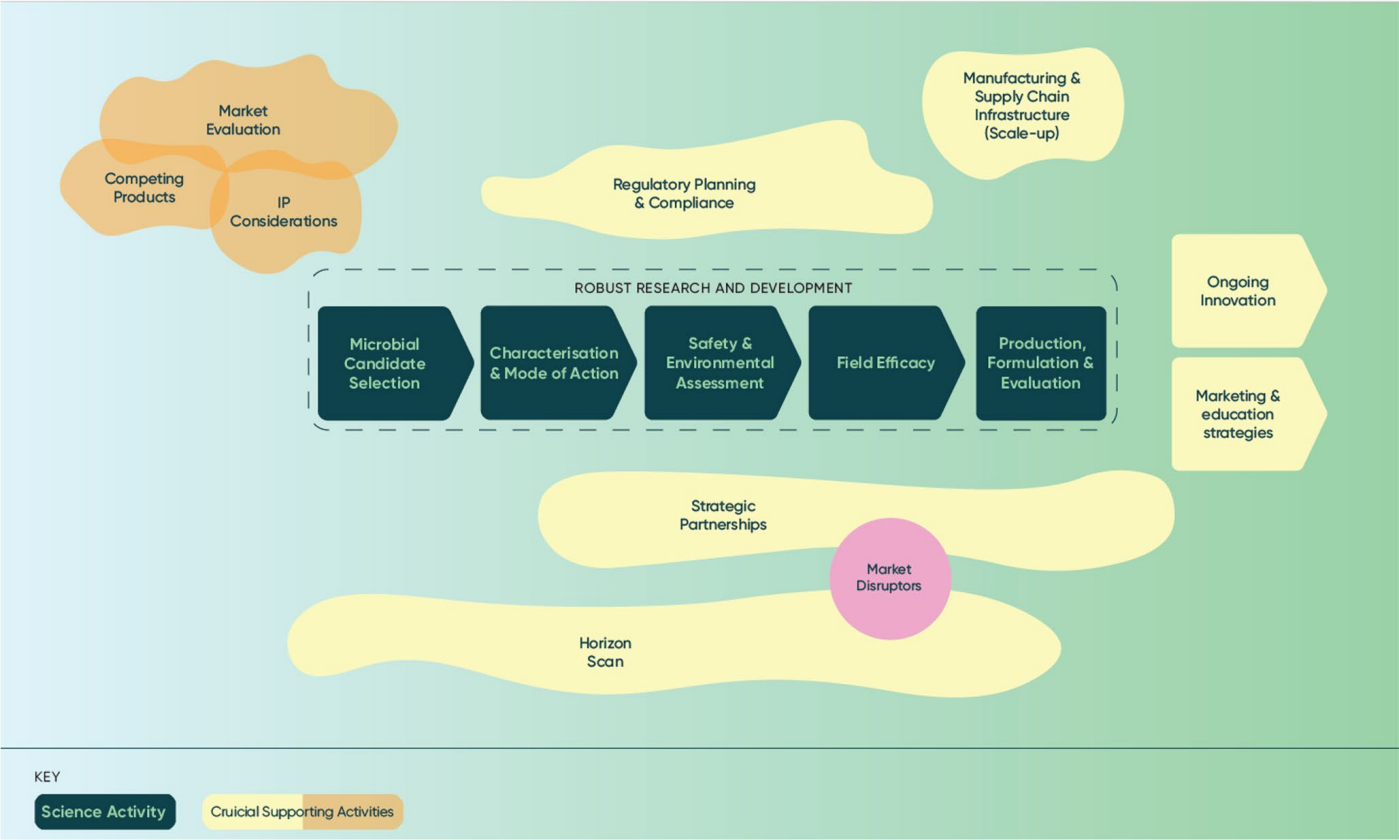
Factors underpinning successful commercialisation and uptake of biopesticides. These include market evaluation, research and development to address science and technical issues and wider management and supporting activities which typically receive less focus and resources than research and development activities but are no less crucial to delivery of products to growers

As discussed above, many literature reviews and industry-generated documents have highlighted the key constraints to development of biopesticides, but problems remain, with large investment made in research on prototypes that will never become products. As portrayed in Fig. 2, there is a mix of both science and technical issues, and wider market and management factors that must be considered for successful commercialization and uptake of biopesticides. Some of these factors have been extensively reviewed elsewhere (Koul 2011; Arthurs and Dara 2018; Ayilara et al. 2023; Nunes et al. 2024) so here we focus on critical points/factors where many biopesticide prototypes have failed. While the usual practise is to consider many of these factors throughout and nearing the end of the development pathway and then reacting to problems encountered during development, these factors must be considered early, i.e. at the start of a targeted development project. Many of these factors can be predicted by early consideration, with either a clear development pathway to overcome issues or taking the decision to abandon the project.

## Establishing the need – know your market

Biopesticide development has often commenced on the assumption that a market exists, without sufficient analysis to substantiate this. Many programs follow a “science push” approach, creating products before assessing demand, unlike the guidance from Köhl et al. (2011) to evaluate markets first. Researchers typically lack expertise in market analysis and value proposition development, leading to unvalidated assumptions about pest significance and the associated market for a new solution. For successful commercialization, it is essential to understand market dynamics, including market size and “market pull” defined as demand originating from market needs or opportunities that drives the direction and prioritisation of product development (Marrone 2024).

**Fig. 3:**
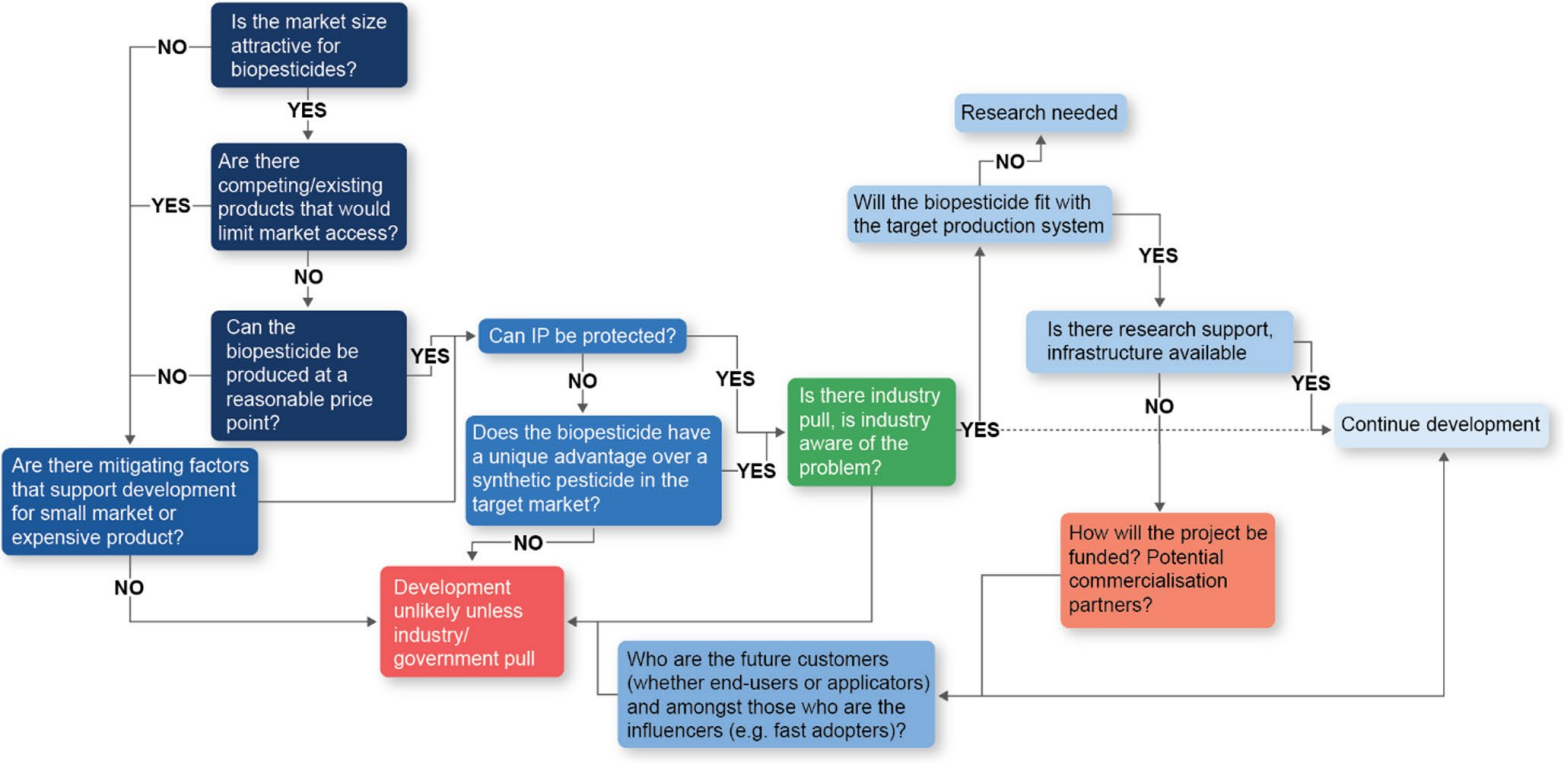
Decision tree for market considerations when developing biopesticides

Market analysis can be hindered by the lack of available data on current pesticide use for a particular pest as this data is often commercially sensitive. Where available, this data reflects current use of existing products and gives an indication of the potential market size at a price point. Investment in thorough market analysis by skilled practitioners can be money well spent and prevents wasted effort and expensive research on a prototype biopesticide that will never be commercially viable. Assumptions about the target market need to be continually tested against the market throughout product development. The lengthy time it takes to get a biopesticide to market necessitates the need to repeat horizon scans for competitors and potential disruptive technologies that may negate the need to develop the product. We suggest key tasks in the market evaluation process in Fig. 3 and discuss below.

## What is the volume or scale of the problem?

Unsurprisingly, many biopesticides developed to date are targeted to pests and diseases that cause yield losses in the most economically significant global crops (e.g. soybean, wheat, potatoes, corn), ensuring large markets. It is more difficult to develop biopesticides that have only regional use or for specific pests on minor crops, as the market size is too small to cover costs of development and registration.

High specificity is no longer a strong driver in the selection of candidate biopesticides because a broader host range increases the potential market size, leading to greater likelihood of commercial success. The various strains and subspecies of *Bt* are a good example of a biopesticide with a relatively wide host spectra, being primarily effective against Lepidoptera, but is also active against species of Coleoptera, Diptera, Hymenoptera, and Hemiptera. One of the key advantages of *Bt* from a commercial perspective is its ability to target over 150 problematic insects, including many important invasive species, such as European corn borer, diamondback moth, gypsy moth, and various mosquito species (Glare and O’Callaghan 2000). *Bt* has also been commercially successful, in part, because it has been the agent of choice to safely manage several invasive forest pests, where high volumes of product have been needed to treat large land areas (Leroy 2025).

Additional future market needs should also be considered, for example crops where it is not possible to breed for resistant varieties, or jurisdictions where the use of genetic modification (GM) solutions are not acceptable, or where the development of resistance to conventional pesticides is leading to a lack of efficacious control options. These scenarios have the potential to extend the market share of existing biopesticide products or present market opportunities for new products.

## Market entry points

Market entry points must be considered carefully. Some growers/sectors are traditionally slow adopters, often because of cost restrictions or the risk of failure to the crop is very high if pest management fails. It may be desirable to initially target niche, high value crops where growers are more likely to follow practices that favour biopesticide use. This enables the demonstration of product efficacy with growers willing to pay a higher cost or having the time to implement a different product into their IPM procedures. A later decrease in cost, for example through improved production methods, enables subsequent entry into low-cost broadacre markets. Some biopesticides have been targeted towards organic producers as early adopters. This market, along with many horticultural producers, feeds into “consumer pull” (demand driven specifically by end‑users’ preferences, expectations, or concerns, influencing which products are adopted or succeed commercially) and the requirement for less pesticides on unprocessed foods. Importantly, there is a need to manage expectations of industry and consumers to prevent frustration and termination of research through unrealistic expectations/timeline (Batista and Singh 2021). But for early consideration, having a clear plan on first clients will assist success.

## What is a reasonable price point for the product?

The starting point for any price point is consideration of existing products used (see below). Markets differ greatly in the amount that can be spent on pest control, from less than a cent per tree per year in long rotation forestry, to high value crops such as some horticultural products. Although it can be difficult to determine what an eventual biopesticide will cost, there are some basic considerations that provide a guideline. The type of organism under consideration will suggest how easy mass production will be, although there can be strain differences within species. Where manufacturing and formulation technology is known to work for closely related microorganisms, this knowledge can be used to provide some estimation of cost of the end product. If the organism is untried at a commercial scale previously, evaluation of the production and formulation approach and likely cost of the process will need to be made.

## Are there any competing products?

Biopesticides have always faced stiff competition from well-established and less expensive synthetic pesticides with putative track records of efficacy and reliability, but this is changing with the slower rate of development of new chemistries and widespread development of pest resistance. The story of the development of *Bacillus thuringiensis tenebrionsis* (*Btt*) and the impact of a new pesticide, imidacloprid, entering the market demonstrates how a biopesticide can be easily replaced by a cheaper competing product (Gelernter 2004). As a Coleoptera-active *Bt* strain, *Btt* was rapidly developed into products for control of the Colorado potato beetle in the USA in the late 1980’s. However, the launch of imidacloprid in 1994, which was more efficacious, eroded the market and almost no *Btt* products remained in the market by 2001.

Tracking the regulatory status of existing synthetic pesticides can allow identification of new market opportunities. Deregistration of relatively inexpensive pesticides is happening regularly in response to environmental and public health concerns, and this can tip the balance in favour of a biopesticide option that previously may not have been able to compete on price or efficacy.

An interesting consideration is how much better a new product needs to be to be to take a significant market share from an existing product? This is a pertinent question which should be addressed early when developing new strains which will compete with an existing microbial biopesticide in the market. For example, there are many existing products based on *Beauveria* and *Bacillus* spp. in the market, but there is still extensive research into new strains of these genera for specific target insects. If an existing product has registration for the target pest, how much more efficacious does a new product need to be to be worth the cost of development and registration? The answer will depend on market size, and any marketable potential benefit.

## Does the biopesticide have an advantage over a synthetic pesticide in the target market?

In addition to demonstrated efficacy, the “story” of the product will be important in achieving market penetration. What will the pitch be for the need for this new product, how is it better than existing products, or how does it fulfil an unmet need? Biopesticides can have a market advantage in terms of product safety compared to many existing products (Marrone 2019; Kumar et al. 2021; Hezakiel et al. 2023) but it is important to understand how the intended product is safer than competing products to define the market edge. There may be issues with the current control options that create opportunities for biopesticide, e.g. restrictions on the number of synthetic pesticide applications to meet minimum residue requirements for export crops or lengthy withholding periods before re-entry and/or harvest of crops that can be reduced by use of a biopesticide.

## How will the biopesticide fit with the target production system?

It is essential to understand the lifecycle of the target pest/disease and identify the window of opportunity for the biopesticide. Will it be used to replace current control measures or be used as part of an integrated pest management solution? Some consideration of how an agent might eventually be distributed and applied in the field can be useful. Chemical pesticides have an established and rich support network to assist users to apply them effectively and at the right time to achieve optimal impact. In addition, many decades of research and development in boom sprayer equipment and training, nozzle designs, adjuvants, and other additives have optimised spray droplet size to achieve consistent field efficacy of chemical pesticides, usually defined as pest mortality. In contrast, biopesticides can act in fundamentally different ways and may need to be applied repeatedly and under different environmental conditions to chemical pesticides. However, if novel and not readily available application equipment, or establishment of a new supply chain is needed, it is far more difficult to get uptake.

## Is the current and future regulatory environment favourable?

When considering the potential market, it is also important to clearly understand any regulatory requirements and the agencies responsible for regulatory compliance. As outlined above, many countries/regions have enacted legislation to encourage use of sustainable pest control options, including biopesticides. This also includes increasing taxes on synthetic pesticides. Such legislative push may alter the “who wants this?” question, by reducing the availability of competing products, increasing government support or subsidiaries, or easing the regulatory burden on new products. However, it is worth noting that not all such legislative efforts have led to increases in biopesticide use (Marrone 2024). For example, the French Government in 2008 called for pesticide use to be halved and spent nearly half a billion euros on implementing the “Ecophyto” plan. However, rather than decline, pesticide use had increased by 12% by 2018 (Stokstad 2018). The recent rejection by the EU parliament of the “Green Deal” proposal, to halve chemical pesticide use by 2030, also shows how fragile any reliance on government level interventions can be (Pollex 2025) and highlights the need to keep abreast of changes in regulatory regimes in jurisdictions relevant to the target market. It is also important to note that regulatory challenges for the efficient risk-based approval of biologicals as compared to synthetics remain in many jurisdictions (Arora et al. 2016; Desai et al. 2016; see below).

## What happens if the market is small?

In some cases, the commercial market may be small or regionally restricted but there is a significant need, for example, to address a biosecurity incursion where synthetic pesticide use is not desirable (treating urban areas or natural estate), or where an export market access is threatened by failure of current pest control measures, or there may be indirect public benefit. These scenarios usually require government support. For example, in some areas, particularly North Africa, mosquito control in areas of high malarial occurrence has been funded by governments or non-government organisations (e.g. The Global Fund, Gates Foundation, USA Malaria Initiative- PMI, Wellcome). Such support changes the potential to develop a biopesticide for a small market.

A highly specific microbial biopesticide that is active against a single pest, particularly an endemic pest, presents large challenges to commercialization and will likely only succeed in niche markets and situations where there is compelling need. For example, the entomopathogenic bacterium *Serratia proteamaculans* AGR96X is active against just two closely related endemic beetle species that are economically significant pests of pasture, but only in New Zealand (Hurst et al. 2023). Because of the pending withdrawal of all currently used persistent organophosphates and the significant contribution made by the grass-fed meat and dairy sectors to New Zealand’s GDP, there is a clear but small market for this bioinsecticide, which is currently progressing towards registration. In this scenario, no multinational company will invest in development and commercialization, and the solution will only be achieved by finding the right local partners and working closely with industry groups to support uptake and adoption of the product. This contrasts with the microbial biofungicide Actinovate based on a *Streptomyces lydicus* WYEC 108 strain. Actinovate is registered in several countries for the suppression or control of a broad range of soil-borne and foliar diseases across multiple crops.

## Can IP be protected sufficiently to protect first investors?

Many biopesticide prototypes are developed in government-funded research institutes and universities, where protecting the developed IP is not the first consideration. However, having some type of protection, including patenting or strong trade secret processes for production, increases the likelihood for industry to commercialize. Commercialization is often the best approach to getting a biopesticide used, but companies that invest the considerable funds to develop and register products must be convinced they can maintain market share. In terms of selection of an agent to develop into a biopesticide, the main IP consideration is “can the eventual product be protected from copycat products?”. Hence, ability to protect IP in some manner is an early consideration in biopesticide development (Fig. 3).

This is not the only approach to successful biopesticide development. For example, a range of products based on *Beauveria bassiana* are sold around the world, where the protection is simply the registration data pack on the strain and any production and/or formulation trade secrets. There are also a range of initiatives which had a public good aim rather than commercial. For example, the “Toothpick project” in Kenya, is a farmer-produced a bioherbicide based on *Fusarium oxysporum* f.sp. *strigae* to combat witchweed (*Striga hermonthica*). The project developed a system for distributing the fungus on inoculated toothpicks to farmers who could produce the biocontrol agent on cooked rice (Baker et al. 2024).

## Additional a priori considerations

While it may not be possible to quantify all of the above factors at the commencement of a biopesticide R&D program, deep consideration of the potential market may, in some instances, suggest projects should end early and save valuable resources, or perhaps to not commence. In addition to market size and costs, there is also a need to identify:

- Who are the future customers (whether end-users or applicators) and amongst those who are the influencers (e.g. fast adopters)?
- Who are the potential strategic and commercialisation partners?
- Are there potential sophisticated investors (angel or green investors) and industry support opportunities?
- Are there any obvious impediments to be registration (e.g. mammalian and non-target safety)?

These factors are considered in more detail below.

## Robust and targeted research

**Fig. 4:**
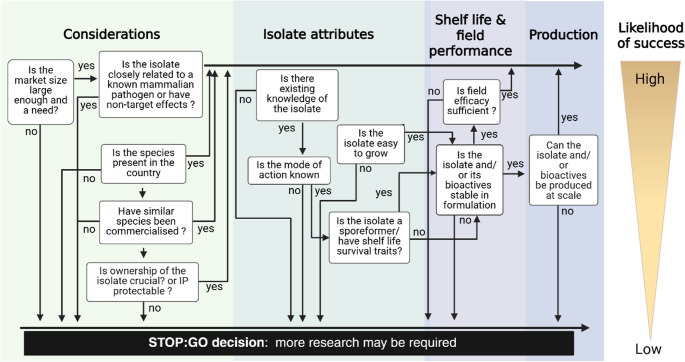
Decision tree, including key stop-go decision points in the biopesticide development pathway

The research approach for developing biopesticides has been addressed in many reviews (e.g. Syed et al. 2018, Vero et al. 2023; Tadesse Mawcha et al. 2024), so our treatment will be brief and focused on how research can be better targeted to expedite and increase successful product development and uptake. All research should be focussed on collecting the right information to enable stop-go decisions needed to prevent wasted investment and effort. These stop-go decisions are predefined evaluation points to assess whether development will proceed, be redirected, or terminated based on evidence and criteria. A flowchart of the main questions that literature and research evaluations should address are presented in Fig. 4.

Köhl et al. (2011) provided useful guidelines and decision-making criteria for stepwise research and screening of microorganisms to be commercialized for control of plant-pathogenic fungi, but the recommendations are broadly applicable to other biopesticidal microorganisms. Selection of optimal candidate biopesticidal isolates will often involve mass screening of multiple isolates, which is now a strength of many companies engaged in biodiscovery and commercialisation. Their access to very large microbial collections and high throughput standardised bioassay systems, together with relational databases and analytical tools is expediting the discovery of a broader range of biopesticidal modes of action. The use of machine learning or AI tools to help select agents early, without extensive laboratory screening, will also reduce development costs.

## Taxonomy, origin of candidate and mammalian safety

Specific identification of the agent to be developed is the first step in evaluation of the ease of potential registration. Microbial taxonomy is constantly changing with reclassification of genera and species happening at an increasing rate, but it is now relatively rapid and inexpensive to identify and classify candidate biopesticide strains (unless it turns out to be a new species). Preliminary taxonomic identification can provide an early indication of whether a novel species/strain is likely to raise any concerns with respect to potential mammalian toxicity or non-target issues (such as an entomopathogenic microorganism having some plant infection ability or phytotoxicity), allowing these strains to be discarded early in any screening process.

One of the first areas to consider must be mammalian safety. Generally, any species linked to a human or animal disease or toxicity will be challenging to register as a biopesticide. Any microorganism that fails a standard Tier 1 safety testing regime should not be pursued. As with all pesticides, human toxicity is of primary concern. A detailed description of the testing of microbial biopesticides with respect to human health has recently been compiled by Wend et al. (2024).

Genomic information assists in early identification of potential non target effects, e.g. potential to produce mammalian toxins. The very widely used bioinsecticide *B. thuringiensis* (*Bt*) is considered safe because of its long history of use with no adverse effects (Raymond and Federici 2017) but given the taxonomy of the *B. cereus* group in which it belongs, it would be complicated to register if it was identified today. Regulators have frequently raised questions about the safety of *Bt* due to its close genetic relationship to several human pathogens or toxin-producing strains, but the taxonomy of the group is now not considered to be related to the toxicity profiles of the strains (Biggel et al. 2022). We have also encountered difficulty with regulators from proximal taxonomic relationships with other mammalian disease-causing microbes, but these can be overcome.

It is important to know how novel a candidate strain is, or if it is exotic or indigenous to the area where it will be used. Indigenous organisms are usually easier to register than microorganisms of exotic origin and will potentially be more suited to the local environment. Where the microorganism is non-indigenous, i.e. where a strain is imported from another jurisdiction because it shows potential against a target pest, the presence of the species in the country of future use can greatly simplify the registration and acceptance for many countries (Fig. 4). Any simplification and shortening of the registration procedure reduces the costs and should be part of the early consideration. The increased availability of global datasets may aid this process as it may be possible to claim global ubiquity (Rodrigues et al. 2017). It is worth noting some jurisdictions do not accept metabarcoding or metagenomic data as proof of presence of a species in country.

There is increasing recognition of indigenous ownership of fauna and flora, addressed in part by agreements such as The Nagoya Protocol, an international agreement signed in 2014 on access and benefit-sharing arising from the utilization of genetic resources in a fair and equitable way (Colella et al. 2023). There are also international treaties such as the Convention on Biological Diversity and the United Nations Declaration on the Rights of Indigenous Peoples. It is important to consider that candidate strains may be subject to indigenous ownership, requiring consultation and agreements around use. An indication of where indigenous rights around organisms is heading can be found in the pharma industry, where several agreements have been reached between indigenous groups and commercial companies (Lal et al. 2024). Consideration of indigenous ownership should be early in a development pipeline, as it can result in difficult and long negotiations if not addressed.

## Mode of action and host range

Whilst targeting promising candidate agents early in the development process is important, targeting a mode of action that fits the needs of a specific market is also critical. One of the compelling benefits of using biopesticides such as entomopathogens, plant-associated microbes or disease-controlling microorganisms, is their unique, complex, or multiple modes of action (MOA), which can offer increased target specificity and reduced risk of resistance development, and sometimes additional production benefits like shorter withholding periods. While information on MOA and host range will require research and does not fit into our treatise of early decision-making, knowledge of the following can help guide research focus.

*How specific is the organism?* Activity of biological agent is often specific to a single group or species of pathogen or pest, and this specificity indicates that most microbial biopesticides do not naturally affect non-targets such as beneficial insects, including predators or parasitoids of pests (reviewed in Ayilara et al. 2023 Chaudhary et al. 2024). While bioactives derived from these microbes may also exhibit host specificity, for example *Bt* toxins (Ragasruthi et al. 2024), it does not imply that this is the case for all their bioactives. In general, negative impacts on non-target beneficials are more likely to be seen when the biopesticide is based on bioactives, rather than formulations of whole organisms (Lisi et al. 2025). Several recent reviews discuss biopesticide MOAs encompassing antibiosis, competition (space, nutrients), parasitism and induction of plant defence (Köhl et al. 2019; Dow et al. 2023). These are not the focus of this review; instead, we detail here consideration of biopesticide MOAs for understanding and evaluating biopesticide efficacy and how this should inform design of field evaluation protocols and standards.

*Is bioactivity due to*,* or associated with*,* exported compounds or metabolites?* Understanding biopesticide MOAs is important for both registration and bioproduction purposes. Even with infective modes of action, exported compounds from microbial biopesticides may be involved in pesticidal activity, so production systems which only harvest the microorganism and don’t include the growth medium in the final product may show reduced efficacy. Conversely, the presence of compounds or metabolites in the final product can also be an impediment to registration, especially if they have any history of toxicity to non-targets, as some regulators will require information on each active. For volatile bioactive compounds, understanding their volatility and water solubility are also important considerations (Belt et al. 2026). The above may appear obvious aspects to understand, but these can be overlooked or unknown until well into the development process.

The use of modern tools, such as genomic analyses pipelines, can greatly assist early decisions on the most appropriate agents for development. The reduction in the cost of whole-genome DNA sequencing has enhanced the cost-effectiveness of synthetic biology and strain engineering. This is supported by the optimisation of predictive algorithms for genome mining of biosynthetic gene clusters, such as antiSMASH – a powerful tool used to detect gene cluster regions responsible for the biosynthesis of secondary metabolites, including terpenoids and polyketides (Blin et al. 2025). These computational tools allow researchers to predict metabolic pathways from genome sequences using manually curated databases like MiBIG (Zdouc et al. 2025). This capability allows for the screening of metabolic pathways potential from isolates in collections as well as from genomes generated through metagenomics sequencing, including metagenome-assembled genomes (MAGs) from environmental samples.

*Will aspects of the mode of action be challenging for a successful biopesticide*? It is important to understand if the biopesticide active form (live or dead microbe, bioactive fermentation extract and/or compound) kills, impairs or repels, and does it work through direct contact or need to be ingested by the pest, or need to parasitise or suppress the pathogen to have effect. This will impact speed of effect, prevent or limit pest or disease activity, and will influence stability needs (UV, thermal) and the impact of environmental factors on product performance. For endophytes or microbial biopesticides that form associations with plants, it is important to understand host specificity (if a limitation), its ability to colonise, and its persistence. The ability to thrive in the plant microcosm is needed for the application of plant-associated biopesticides and more applicable to candidates for biological control over application of their bioactives alone. It may result in ongoing production of the bioactive(s) and achieve further benefit from biopesticides that activate plant defence responses that supress pathogen or pest attack, locally or systemically. Ideally, the biopesticide would act systemically through the plant, resulting in application only needing to reach a plant and prolongs/extends efficacy, but this very rare in any biological agent (Flors et al. 2024).

Biopesticides can act in fundamentally different ways to conventional chemical controls (Table 1). For example, they may not cause direct mortality of insect pests but instead reduce feeding on crops or reduce fecundity over time. They may not stop disease but may reduce disease expression or pathogen inoculum buildup or carryover between cropping seasons. They may act rapidly or be slow to kill. This means field protocols relevant to the biopesticide need to be developed and implemented, rather than a scenario where they are evaluated against and using protocols developed for chemical controls, leading to their inappropriate evaluation and use on-farm (O’Callaghan and Mansfield 2026).

**Table 1:** Crop protection efficacy endpoints and selected examples of research studies that have demonstrated these impacts in relation to biopesticides. Matching the species agent and mode of action (MOA) to need within a market is critical for early decision-making. The endpoints measured (that result from the MOA, if known) in the research phase must reflect the functional outcome that farmers require. Early decisions about what functional outcome farmers require stems from a thorough understanding of the market needs

Endpoint	Target pest/disease issue	Biopesticide impact	Citation (examples)
Reduces disease symptoms, incidence or rate – disease scores/ratings and timing	Fungal pathogens – numerous. Example *Fusarium pseudograminearum*	Two *Streptomyces* isolates lowered disease severity when delivered as seed coats.	O’Sullivan et al. 2021
Reduces disease symptoms – competes with pathogen colonisation	*Botryis cinerea*	*Aureobasidium pullulans* completes for space and prevents *Botrytis* from colonising fruit surfaces.	Reviewed in Di Francesco et al. 2023
Reduces disease symptoms – inhibits pathogen spore germination or development	Fungal pathogens – example *Verticillium dahliae*	Fermentation extract from a *Streptomyces* strain inhibited spore germination, and mycelial growth and branching.	Gallart et al. 2025
Reduces disease symptoms – enhances plant defence responses	Fungal pathogens – example *Rhizoctonia solani*	Microbial extract from a *Streptomyces* strain induced plant defence gene expression and reduced disease severity.	Belt et al. 2021
Reduces pathogen inoculum buildup – in planta foliar	*Sclerotinia sclerotiorum*,* Ascochyta rabiei*	*Streptomyces* derived biofungicide sprays suppressed in planta sclerotia or conidiospore development.	Thatcher et al. 2025 Bullock et al. 2026
Reduces pathogen inoculum buildup – in planta roots	*Fusarium pseudograminearum*	Two *Trichoderma* isolates suppressed *in planta* pathogen inoculum.	Stummer et al. 2022
Reduces pathogen inoculum carryover - viability of overwintering structures	*Sclerotinia sclerotiorum*	*Coniothyrium minitans* infected sclerotia are unable to generate apothecia or produce mycelium of *Sclerotinia* spp.	Reviewed in Sharma et al. 2025
High mortality of adults – direct toxicity effects	desert locust *Schistocerca gregaria*	Entomopathogenic fungi, *Metarhizium anisopliae*, an isolate of *Beauveria bassiana.* Lethal doses caused mortality of adults, sub-lethal doses caused reduced reproduction and slow growth of offspring.	Wakil et al. 2022
	Rhinoceros beetle in oil palms *Oryctes rhinoceros*	Oryctes virus.	Huger 2005
	Diamondback moth, *Plutella xylostella*	Entomopathogenic bacterium *Yersinia entomophaga.*	Hurst et al. 2011
High mortality of immatures or certain life stages – direct toxicity effects	Rhinoceros beetle in oil palm *Oryctes rhinoceros*	The fungus *Metarhizium anisopliae* is developed as biopesticide against immature stages and may be distributed by adults.	Bedford 2014
	NZ grass grub and manuka beetle in pasture	Entomopathogenic bacterium *Serratia proteamaculans*, lethal doses cause mortality of soil-dwelling larvae; as efficacious as currently used synthetic pesticides.	Hurst et al. 2018
Reduced fecundity or reproductive potential	*Spodoptera frugiperda*	Two *Beauveria* isolates prevented reproduction by infected females.	Apirajkamol et al. 2023.
Sterility after mating via CI or SIT	Mosquitos	*Wolbachia* used as a biopesticide. *Wolbachia*-induced conditional sterility, cytoplasmic incompatibility, and the repeated release of incompatible males.	Ross et al. 2020 O’Connor et al. 2012
Deters oviposition on plants	*Helicoverpa* spp.	*Clitoria ternatea* fractionalized extract mixture reduced oviposition and was a feeding deterrent.	Mensah et al. 2015
Reduces mobility or prevents movement in some way	Colorado potato beetle, *Leptinotarsa decemlineata*	Low doses of double-stranded RNA (dsRNA) insecticide (Calantha^®^, active ingredient ledprona) reduced adult mobility, movement between fields.	Pallis et al. 2023
Reduces virus/disease transmission by vectors	Pest aphids on crop plants	Endosymbionts used to alter the vectoring of virus by aphids.	Sanches et al. 2023
Increases the susceptibility of pest to conventional pesticides	Stored grain pests, *Tribolium castaneum*, *Oryzaephilus surinamensi*,* Rhyzopertha dominica*	Combination of graphene and spinosad significantly increased mortality when compared to either substance alone. Might be due to graphene’s ability to enhance contact with the insect cuticle, facilitating better penetration of spinosad.	Lampiri et al. 2025
Increases the susceptibility of pest to biological control agents (e.g. parasitoids and predators)	Resistant strain of the mosquito *Culex quinquefasciatus* (aquatic example)	Exposure of a resistant strain of a mosquito to *Bacillus thuringiensis israelensis* (Bti) caused more direct mortality and predation from the pygmy backswimmer *Plea minutissima*.	Delnat et al. 2019
Alters the thermal tolerances of a pest species.	*Acyrthosiphon pisum* (aphid)	*Beauveria bassiana* affects upper and lower thermal tolerance of aphids and a predator.	Porras et al. 2021

^1^ As shown in Fig. 4

The unique or complex modes of action described in Table 1 can pose both drivers for and barriers to adoption, so understanding how these novel MOAs address the needs of the target market early in the biopesticide development process is essential. For example, do new methods need to be developed to measure a novel or slow acting MOA compared to more common and well-studied MOAs with efficacy endpoints such as pest viability scores or visual disease symptoms. Even with the latter, if the disease scores are variable or not amenable to high-throughput phenotyping, it may delay or inhibit development of the biopesticide or impair the ability to market the product. Biopesticide agents that compete with pathogen colonisation on planta or deplete pathogen inoculum in the field may require detailed time course studies (potentially over years) to show benefit. In related examples, microbial bioinsecticides such as *Lecanicillium lecanii*,* Isaria* sp. and *Beauveria bassiana* have the capacity to provide good control of whiteflies under field conditions, but they have slow speed to kill compared to chemical controls (Sharma et al. 2023; Sani et al. 2020). For other biopesticides the speed of kill is too slow to be used in a commercial agricultural setting such as for some baculoviruses of caterpillars that have a long infection period, 10–18 days, that allows significant damage to the plant before pest death (Grzywacz 2017; Kour et al. 2025). This can be considered early in the biopesticide development process for some organisms with well-described MOAs. Rapid mortality of large numbers of insect pests as they reach critical or economic thresholds is not feasible for biopesticides that work slowly, something that needs to be factored into early market size considerations (Fenibo and Matambo 2025). In scenarios where knockdown effects are required, an insecticide with strong contact activity could be used, followed by a biopesticide product that effects insect feeding or development for example (Marrone 2024). The downside of this approach for a biopesticide product is that the follow up product may not be needed if the pest population is controlled, affecting the size of the market.

## Environmental safety and effects assessment

The biosafety of biopesticides is one of their key advantages as pest management tools and this is increasingly true as the rate of withdrawal of synthetic pesticides increases in response to public concerns around human health and non-target effects on the environment. While biopesticides are generally considered as environmentally benign alternatives to broad spectrum pesticides, they are not entirely without risk and multiple reviews have addressed techniques for, and challenges in, assessment of the biosafety of biopesticides (Hokkanen and Hajek 2003; Borges and Mendelson 2025). Data is often available early in development projects about mammalian and environmental safety, so can be used to select agents. Hazard testing forms the basis of most risk assessments for biopesticides with Tier 1 hazard tests designed to represent worst case exposure scenarios and higher tiers progressing through more complex testing that mimic increasingly realistic conditions. In addition to mammalian toxicity, additional Tier 1 non-target organism testing requirements includes high dose testing on birds, freshwater and marine fish and invertebrates, non-target plants and invertebrates, including honeybees. Depending on the jurisdiction, there may be additional requirements to test iconic, endemic or culturally significant species that are closely related to the target pest. Mortality is usually the endpoint of this testing and if mortality is not observed, testing can be concluded. If needed, subsequent testing in higher tiers includes collecting data based on chronic and wider host range testing. In their overview of guidelines and methodologies for non-target organism testing, Karaoglan et al. (2024) highlighted that there is still significant work to be undertaken to ensure globally consistent and robust evaluation of the biosafety of biopesticides, work that will require collaboration between many different parties, stakeholders, and regulatory agencies across borders.

The very characteristics of biopesticides that are often regarded as disadvantageous with respect to commercialization – host specificity (leading to a limited market) and short environmental persistence (which can reduce shelf life and field efficacy) - underpin their biosafety, but host range varies widely. Most viruses developed for pest control are baculoviruses and because of their high specificity, no negative ecological effects have been documented (Cory 2003). There is growing interest in the use of bacteriophages as control agents for bacterial plant diseases, with several products on the market (Czajkowski et al. 2025). These too will likely have few non-target effects given their very high specificity to pathogenic bacterial species/strains. The more broad-spectrum biopesticides warrant closer scrutiny of their biosafety beyond mammalian toxicity and phytotoxicity and evidence must be gathered to show that there are no effects on a wide range of other organisms including beneficial non-target species such as pollinators, earthworms and other soil organisms, including microorganisms, biocontrol agents (predators and parasites) and, if appropriate, aquatic species.

In large-scale eradication or control programs, vast areas may be treated repeatedly, making the environmental safety of the agent crucial. This is increasingly important as treatments can occur in urban areas, ports, and other sensitive locations. Robust, balanced data on environmental and mammalian impacts of biopesticides is therefore essential for regulators and the public (O’Callaghan and Brownbridge 2009).

Genetic analysis presents a significant opportunity to expedite and streamline risk assessment processes early in the development pathway. Whole genome sequencing of biopesticidal strains is now a key part of the identification of new isolates, alongside classical phenotyping approaches. Genomes can be rapidly screened for the presence of biosafety “red flags” e.g. genes indicating the potential to produce mammalian toxins and as sequencing ability increases, the number and types of traits analysed will increase. Research on the genetic basis of pathogenicity and virulence factors expressed in pesticidal microorganisms is contributing to understanding of infection processes, toxin production and specificity, which can potentially be used to predict possible non-target effects. Currently, this research is restricted to a small number of well characterised microorganisms and their associated hosts for which comprehensive databases exist, but rapid advances in technologies, including in silico approaches, should expedite risk assessment in the future.

## Production and formulation

*Can the microorganism or its bioactives be produced at an economically feasible price?* The microbial active must be able to be cost-effectively mass-produced as viable, efficacious, and genetically stable propagules. Lack of affordable production systems often prevents commercialization, sometimes after significant investment. Microbes that cannot be readily cultured or are genetically unstable are poor candidates. However, ease of culturing the laboratory does not guarantee low-cost scale-up, which is where challenges often arise.

Bacteria are typically cultivated using liquid fermentation, while fungi either liquid or solid state fermentation (Verma et al. 2024). Liquid fermentation in large fermenters offers lower batch costs but requires high capital investment. Solid-state fermentation, common for Ascomycete entomopathogens like *Beauveria* and *Metarhizium*, is cheaper to set up but labour-intensive. The choice of approach will be dictated by each product type and regional issues, such as availability of contract manufacturer, industry partner and labour costs. The key question that must be asked prior to a development project is whether mass production is feasible at a realistic cost? It is easy to calculate the predicted cost of a potential product based on cost of growth media, but that is only part of the cost of commercial production. Costs need to include depreciation on equipment, labour, a company margin (no industry survives if they are simply covering their costs) and other incidentals such as power.

Any downstream processing also needs to be considered, both from the viewpoint of feasibility and added costs. Not all processes used in a laboratory are feasible at an industrial scale. Any process added after the fermentation step such as drying, extrusion, or seed coating requires equipment and labour, further increasing costs, but may reduce the cost of application. For example, the use of seed coating may enable its effective application via seed drill and in the vicinity of a pests of the applied seed (O’Callaghan 2016).

Research should target low-cost media options and seek to reduce growth times while maintaining yield, stability and efficacy, rather than simply focussing on producing high biomass. This could be achieved, for example, through process optimisation, metabolic modelling or domesticating strains towards growth on cheap feedstocks.

Formulation for environmental persistence and product shelf-life has also been identified as a critical step (Muskat 2025) and the lack of suitable formulations has led to several orphaned biopesticide technologies (Boyetchko 2020). Early consideration should be given to how a stable product will be achieved. For example, is the organism a spore-former, which is much more likely to be shelf-stable, or will novel formulations need to be developed?

Biopesticides are typically applied to an environment different from where they were isolated from or adapted to, potentially reducing their ability to compete with the resident microflora and affecting their environmental persistence. This may necessitate that a developed formulation increases the stability but also imparts a competitive advantage for the applied microbe post application. It has been assumed that industry could fill the void in knowledge in formulation technology, with many companies having in-house expertise in this area. However, excipients used in chemical pesticide formulation development are not necessarily compatible with microorganisms and scaled production and formulation of shelf-stable microbial products remains a significant hurdle (Santos et al. 2021; Marrone 2019; Zhang et al. 2018). Biopesticides in the form of spore-forming microbes like *Bt* and Actinobacteria are inherently more stable than non-spore formers. For example, Actinovate products based on *Streptomyces lydicus* have a shelf life of two years from the date of manufacture when stored under standard warehouse conditions, meaning no refrigeration or special handling is required (Novonesis 2026).

It is important to define the most cost-effective delivery method that will be compatible with available technologies at the time of product launch. Application technologies may comprise the use of an inert carrier formulated as a granule with ballistics that allows optimal distribution, or the ability to be drilled, or used as a spray, or seed coat. Each of these technologies require different manufacturing routes, specialised equipment each with their own associated production and application costs. A dried product may be less expensive to transport than its aqueous equivalent. Post application biotic variables such as seed, or plant type may impact on microbe stability, while at the same time an applied microbe will need to effectively compete with the resident soil/plant microflora (Copeland et al. 2025). These variables require an efficacious and cost-effective formulation. While solutions to these potential issues may not be available at the start of the development process, solutions will be required and need to be identified and tackled early in the development process.

In high value crops, costs may be reduced through use of advances in pathogen/ pest sensing and precision application (i.e. robotics, drone technologies), enabling the agent to be applied at the correct time and location (Lochan et al. 2024; Padhiary et al. 2025). Precision applicators have the added advantage of reducing the active exposure to environmental stresses. Costs may be incurred through need for specialised storage of the formulated active through the supply chain, but refrigerated storage is becoming increasingly accepted if it maintains the efficacy of the product, e.g. ryegrass seed endophytes (Hume et al. 2011, 2013).

## From greenhouse to field efficacy trials

Establishing proof of concept (PoC) and conducting grower-led validation field trials are critical steps in the development and adoption of novel biopesticide products for high-value and broad acre crops and are not areas that are easily predicted from minimal data, so we provide less focus on this area in this review. The PoC phase typically begins with laboratory and greenhouse experiments to assess efficacy, optimal dosages, timing and method of delivery, and modes of action under controlled conditions, often using a range of crop varieties to identify genotype-dependent responses and compatibility with the biopesticide. These early trials should also evaluate product stability, shelf-life, and environmental persistence, as biopesticides often have shorter residual effects compared to synthetic pesticides, necessitating repeated applications or improved formulations for field robustness (De La Cruz Quiroz et al. 2019). Iterative rounds of formulation development can be undertaken to improve product stability and on planta efficacy. It is common for microbial agents that look promising in laboratory and glasshouse pot trials to perform less well when applied in the field (e.g. Smyth et al. 2011), so field screening of potential microbial candidates sooner rather than later in the development process can be warranted to avoid investment into less promising candidates.

Once laboratory and greenhouse efficacy is established, small-scale field trials should be conducted under diverse environmental conditions to simulate real-world scenarios, focusing on pest/pathogen control efficacy, crop safety, and integration with existing agronomic practices (Clerc et al. 2025). Correct application must be informed by understanding the biopesticide’s MOA and how that relates to the efficacy endpoints that need to be measured in trials (Table 1). Most biopesticide field trials aim to determine the efficacy of prototypes or commercial products against an untreated control and the current industry standard treatment, usually a synthetic pesticide, but sometimes a commercially available biological product. Inclusion of a “formulation blank” control (application of product containing all formulation ingredients but without active microorganism(s) is desirable but not always possible when the full details of a commercial formulation are not disclosed (Bashan et al. 2020). Failure to include this control treatment can result in “unfair” comparisons between an unformulated prototype microorganism against commercial products with formulations optimised for extended field persistence, adherence to foliage, UV protection, etc. Trial design may also need to consider other variables such as correct storage and handling, application timing (on day of application and through crop growing season), environmental factors (e.g. temperature, humidity), and soil properties, all of which can influence biopesticide performance. This information is critical for growers to understand that, unlike chemistries, biopesticide efficacy can be impacted by the environment and are most often not a silver bullet.

Further into the development process, for grower-led validation, collaboration with commercial growers, their agronomists or advisors, and with product manufacturers is essential. Growers should be involved in trial design, product application, and data collection to ensure practical relevance and foster adoption (Akutse et al. 2020). Demonstration sites, conducted on farm and in collaboration with sector groups are a useful extension tool to publicise results and broaden adoption. Extension services and public-private partnerships can facilitate training, knowledge transfer, and feedback loops, helping to refine application protocols and address barriers to adoption such as product reliability, cost, and regulatory hurdles (Acheuk et al. 2022). Ultimately, successful PoC and grower-led validation requires iterative, multi-location trials, transparent communication among stakeholders, and a focus on both agronomic efficacy and economic viability to support the transition from experimental product to commercial adoption in high-value and broad acre crop systems. This points to early engagement with growers and other strategically important partnerships.

Field trials allow collection of efficacy data necessary for registration but if well designed can also demonstrate additional benefits beyond management of the target pest or disease. Some additional benefits can be inferred from knowledge of the agent prior to field trials and thus contribute to decision making early. Field trials can demonstrate how a novel biopesticides can be integrated with a diversity of other control options for controlling pest populations. Growers are often more interested in consistency in crop protection and crop yield or economic gains rather than pest numbers (Marrone 2007; Chandler et al. 2008; Adeniji et al. 2024), so compelling yield data collected under a range of conditions supports future uptake and adoption. For example, growers may appreciate a product that performs at 50% efficacy in 100% of applications, over a product that has 80–100% efficacy but works only 50% of the time. Consistency in efficacy and knowing how a product works (Table 1) allows growers or advisors to develop IPM plans that may incorporate multiple biopesticide solutions that increase the overall efficacy of their crop protection solutions (Glare et al. 2012).

## Regulation

Biopesticide regulations are region-specific (Table 2) although they share many common characteristics, including that they are often based on the regulation of chemical pesticides. This is a highly complex area with regulations constantly undergoing review and change (Grant and Gwynn 2020), so rather than discuss regulations per se, we focus here on regulatory impediments to successful product development, some of which can be anticipated early in the development project. This requires knowledge of what data will be required. The data requirements of a regulatory dossier generally include:

**Table 2:** Comparison of regulatory requirements across four regions (Frederiks and Wesseler 2019; Andreata et al. 2025; Karamaouna et al. 2023; Kumar et al. 2025; Reddy et al. 2024)

Criteria	Brazil	USA	India	EU
Time in the regulatory process	12–36 months depending on type	~ 2–3 years, expedited review	Often slow; companies can face delays	7–9 years (improving)
Category & review	Recognized as “low risk”	Specific category, “reduced risk”	No separate category; treated as chemicals	Same as chemicals, slow review process
Data demands	Efficacy required; reduced toxicology data	Tailored data, possible waivers	Full toxicology & efficacy; possibly excessive	Full safety, efficacy, environmental data
Cost of regulation	Moderate; multi-agency compliance	Generally lower than chemicals	High burden, chemical standards	High cost, extensive data
Incentives & reforms	Tax/subsidies, support for on-farm use	Fee reductions, EPA support for startups	Guidelines established	EU push for fast-track lanes & a proposed Biotech Act^1^

^*1*^ Proposal for a Regulation to establish measures to strengthen the Union’s biotechnology and biomanufacturing sectors (European Biotech Act)

- Activity agent(s)
- Mode of action
- Non-target (sometimes including phytotoxicity), environmental and human safety aspects
- Persistence/residue levels (MRLs)
- Quality, purity and stability of the product
- Product efficacy for all label claims over a range of geographies
- Manufacturing process
- Shelf life

Collecting data to address these requirements must be a key focus for any research and development project from the start. As stated above, mammalian toxicity should be established as a priority during the research phase. Complete dossier data requirements for mammalian toxicity in the USA and EU were reviewed by Wend et al. (2024). How much data is required is usually region specific, but a sufficient number of field trials can often be difficult due to cost. Host range testing requirements is another area that needs to be considered early in the development process, as toxicity to bees, for example, would make registration difficult for outdoor crops.

There are two main issues often associated with the registration process, the time taken to achieve registration and the complex requirements for registration are often based on chemical pesticides, so can be difficult to conform with for a biological product (Table 2) (Balog et al. 2017). In the EU, biopesticides are not treated as a separate category as they are in the USA, although there is a low-risk category (Karamaouna et al. 2023). Instead, the EU process is slow and involved, with several organisations and member states involved from the 27 EU states and little flexibility. The role of regulation is very important in assuring safety, both environmental and mammalian, but data requirements based on pesticide chemistry can be onerous or unnecessary for biopesticides. The requirement for both EU and then individual country registrations delay the process more than in other jurisdictions. Frederiks and Wesseler (2019) estimated the EU process was on average 1.6 years longer than that in the USA, taking up to 7 years. At the other end of the spectrum, Brazil average is now 1–2 years, and this has contributed to a massive increase in biopesticide registration and use, from below US$58 million in 2017 to US$690 million in the 2023/24 season (Andreata et al. 2025).

## Strategic partnerships for commercialisation

Achieving successful biopesticide commercialization is challenging, even once proof of concept has been achieved and the IP is protected (Kumar et al. 2021). Strategic partnerships are essential to bridge the gap between discovery and market adoption. Having some knowledge of which partnerships will be crucial to product success at the start of the development can greatly improve the chances of a successful biopesticide development project. Partnering early with potential distributors and growers can greatly improve the chances of successful development, by providing reality checks on the putative biopesticide.

As discussed above and unlike synthetic pesticides, many biopesticide opportunities emerge from academic or public research institutions looking for biological solutions to specific national pest and disease impacts, characterised by a localised market need and opportunity (Copping and Menn 2000). Such laboratories often excel in isolating and characterizing new biological actives with pesticidal potential from the biodiscovery programs, conducting bioassays, and elucidating mechanisms of action, but have very limited capacity for, or knowledge of, large-scale production, formulation optimization, regulatory navigation, or marketing. Consequently, partnerships with private industry and investors, and/or with governments providing support under national priorities to move away from synthetics is required to translate promising laboratory discoveries into commercially viable products, often via an SMEs start up model. Effective collaborations enhance access to specialized expertise and infrastructure, distribute financial risk, and align the technical innovation pipeline with market and regulatory realities to help it through the commercial “valley of death”. Identifying these potential collaborations and support networks early can improve success.

The private sector provides the resources and infrastructure for fermentation technology, pilot production, formulation chemistry for stability testing, and compliance with good manufacturing practices (GMP). Availability of such collaborative partnerships also provide the scalability expertise necessary to produce consistent, shelf-stable, and field-effective products. In turn, private partners benefit from access to the IP and scientific validation, generally formalized through licensing agreements, joint ventures, or public–private partnerships.

## Marketing, grower support and education

As mentioned in several sections already, having key partnerships with industry can greatly improve biopesticide development success. Marketing of the biopesticides can be crucial to uptake, with growers often reluctant to change to untried new products unless recommended by trusted advisors. Active participation by grower groups or industry as part of the development team early in the process, therefore, is highly beneficial. Having such participation from industry during early evaluation improves the chances of successful decision making.

Having trained agronomists and experienced and timely support from extension specialists in relation to the application of biopesticides is lacking in many regions. For example, the arrival of fall armyworm (*Spodoptera frugiperda*) into Australia in 2020 (Piggott et al. 2021) led to high pest populations and significant damage to sweet corn fields. Whilst conventional pesticides were available, agronomists quickly realized that the loss of beneficial species, and the likelihood of resistance development, meant biological options needed to be integrated into their systems. An emergency use permit was issued for the use of Fawligen^®^ a selective baculovirus biopesticide product. However, trial and error applications in commercial fields showed that this product needed to be applied early in the corn growth cycle and on first instar larvae (with monitoring needed to target applications) (Brown 2023). Knowledge of the entomovirus literature could largely have predicted this, pointing to early consideration of the agent and how it is likely to operate in the field as part of the evaluation process. Successful integration of biological products and chemical pesticides could be achieved but learning and industry-wide education was required. Importantly, agronomists were at the forefront of the knowledge-sharing and workshops to extend examples of successful control.

Another method that can increase biopesticide uptake is an industry recommendation or requirement, an example of which was the addition of a biopesticide AureoGold into the New Zealand kiwifruit industry recommended treatment list for their growers (Hoyte et al. 2018). In this example the industry body Zespri dictates best practice and incentivised rapid uptake of the biopesticide for management of Psa disease (Scarlatti 2025). Identifying if this is likely early in the process can help guide research and development decision making.

## Ongoing innovation – next generation products and processes

In addition to current biopesticide technologies, research and commercialization of new and innovative technologies can identify new MOAs, and improve target specificity, stability and bioproduction. Coupling these generation 2 products with the decision-making guidelines detailed above, provides a pathway for sustainable crop protection. During the development process, constant horizon scanning is also required, especially for any disruptive technologies that might undercut the market for a new biopesticide. Examples of innovations that are likely to be instrumental in biopesticide success in future years includes genetic engineering, synthetic microbial communities, endosymbionts and endophytes. Each areas brings unique considerations for product development, but essentially requires the same considerations as covered above.

Genetic manipulation which can facilitating the removal of undesirable traits or enhance beneficial traits is already in use. For example, vegetative insecticidal proteins such as those produced by *Bt* can be synthesised to be more effective in host binding or to alter host range, potentially overcoming current limitations for commercialisation, a process achieved through amino acid alteration, domain swapping, and construction of protein chimeras (Gupta et al. 2021; Jiang et al. 2023). The use of heterologous expression systems, such as *Escherichia coli* or *Saccharomyces*, is making the production of resultant metabolites, proteins and peptides scalable (Fletcher et al. 2020; Guan et al. 2021). For example, cell free in vitro expression has enabled the cost-effective production of RNAi, to produce the Colorado potato beetle product Calantha™ (Cedden and Bucher 2025; Mendoza-Alatorre 2025; Narva et al. 2025). However, the same process for market (Fig. 3) and agent (Fig. 4) evaluations need to be applied.

Endosymbionts, host–dependent bacteria that naturally occur in pest populations and support a diversity of functions within their host (Hoffmann and Cooper 2024), and endophytes, microbes that live inside plants, have been widely investigated as agents that can improve pest management. A priori consideration of a potential endosymbiont or endophyte as a commercially available biopesticide would follow a similar process as shown in Fig. 4.

Most commercial microbial products to date are formulated as axenic (single species) cultures, often containing strains from a small rotation of the same microbial candidates. This largely reflects the challenges associated with scaling multi-species cultures (such as microbe-microbe incompatibilities); formulation constraints that escalate in a non-linear manner for every additional microbe; and registration frameworks that are not suited to assessing multi-microbe products. However, an emerging strategy is to embrace microbiome complexity by developing products based on *microbial consortia* (any assemblage of two or more microbial species, whether natural or artificially combined) or *synthetic microbial communities* (SynComs; deliberately constructed consortia, with a composition designed to capture specific functions or interactions), delivering versatility through additive or non-additive effects (e.g. Magnin-Robert et al. 2013; Tienda et al. 2024; Peterson et al. 2024). It is currently very difficult to assess the potential of SynComs a priori, in the manner we are suggesting in this paper due to the lack of studies.

## Conclusions

The development of microbial-based products for agriculture starts with selection of the organism to form the basis of the active agent and proceeds through R&D stages to demonstrate efficacy, safety, and price competitiveness of the final product. It is estimated around 90% of all development projects fail, due to some limitation of the organism, formulation or market. A more rigorous early evaluation of the organism (and/or its active compounds) against the known common barriers to biopesticide product success will reduce the failure rate and increase the proportion of biopesticides that successfully enter the market. As outlined in Fig. 4, by considering the biological organism’s characteristics (including efficacy, environmental requirements, production and formulation characteristics), market need, ability to register for that market and safety early in the process, successful development of biopesticides should increase.

## Data Availability

No datasets were generated or analysed during the current study.
